# Living with a Bullet in Her Brain

**Published:** 1886-02

**Authors:** N. C. Miller

**Affiliations:** Stroudsburg, Pa.


### Living With a Bullet in Her Brain.

*Eds. Medical and Surgical Reporter:—*

On January 10, 1882, a two-year-old child of a Mr. Newhart, while standing in a doorway was accidentally shot by a lady who was carelessly handling a gun. The ball penetrated the right temporal bone just above the centre. The child, uttering a scream, fell unconscious to the floor. I arrived in two hours, and found brain matter oozing from the wound. I probed for the ball, but thinking that it had passed through the brain, struck the opposite side of the skull and glanced off, I desisted from any further attempts at finding it. I kept the child on pot. bromid., and treated the symptoms as they arose. The external wound healed inside of ten days. There was complete paralysis of the right arm and leg, showing that the left side of the brain was injured. At first the child was entirely blind and deaf, but after several months the left eye and ear regained their functions; and within the last year sight and hearing have been restored. On the other side the paralyzed limbs are also regaining their strength, and now, at the age of five, she is for the second time learning to walk. She is taller and nearly as heavy as her twin brother. Her intelligence is somewhat affected, but is improving. She is subject to epileptic fits, but is otherwise, to all appearances well. She has also passed through several severe sicknesses since the injury. N. C. Miller, M. D.

Stroudsburg, Pa.

The University of Basle possesses the oldest known anatomical preparation. It is the skeleton of a human body, and was prepared by the founder of anatomy, Andreas Vesalius. It bears the date of 1543.

